# Multiple QTL underlie milk phenotypes at the *CSF2RB* locus

**DOI:** 10.1186/s12711-019-0446-x

**Published:** 2019-01-24

**Authors:** Thomas J. Lopdell, Kathryn Tiplady, Christine Couldrey, Thomas J. J. Johnson, Michael Keehan, Stephen R. Davis, Bevin L. Harris, Richard J. Spelman, Russell G. Snell, Mathew D. Littlejohn

**Affiliations:** 1 Research and Development, Livestock Improvement Corporation, Ruakura Road, Hamilton, New Zealand; 2 School of Biological Sciences, University of Auckland, Symonds Street, Auckland, New Zealand

## Abstract

**Background:**

Over many years, artificial selection has substantially improved milk production by cows. However, the genes that underlie milk production quantitative trait loci (QTL) remain relatively poorly characterised. Here, we investigate a previously reported QTL located at the *CSF2RB* locus on chromosome 5, for several milk production phenotypes, to better understand its underlying genetic and molecular causes.

**Results:**

Using a population of 29,350 taurine dairy cows, we conducted association analyses for milk yield and composition traits, and identified highly significant QTL for milk yield, milk fat concentration, and milk protein concentration. Strikingly, protein concentration and milk yield appear to show co-located yet genetically distinct QTL. To attempt to understand the molecular mechanisms that might be mediating these effects, gene expression data were used to investigate eQTL for 11 genes in the broader interval. This analysis highlighted genetic impacts on *CSF2RB* and *NCF4* expression that share similar association signatures to those observed for lactation QTL, strongly implicating one or both of these genes as responsible for these effects. Using the same gene expression dataset representing 357 lactating cows, we also identified 38 novel RNA editing sites in the 3′ UTR of *CSF2RB* transcripts. The extent to which two of these sites were edited also appears to be genetically co-regulated with lactation QTL, highlighting a further layer of regulatory complexity that involves the *CSF2RB* gene.

**Conclusions:**

This locus presents a diversity of molecular and lactation QTL, likely representing multiple overlapping effects that, at a minimum, highlight the *CSF2RB* gene as having a causal role in these processes.

**Electronic supplementary material:**

The online version of this article (10.1186/s12711-019-0446-x) contains supplementary material, which is available to authorized users.

## Background

In much of the Western world, milk is primarily produced for human consumption by taurine cattle (*Bos taurus*) dairy breeds. Within these breeds, many generations of selection have improved milk production capacity and efficiency. However, in spite of numerous recent genome-wide association studies (GWAS) e.g., [1–4], major QTL remain for which no causative gene has been definitively assigned.

Several genes with substantial impacts on milk yield are known, including *DGAT1* [5], *ABCG2* [6], *GHR* [7], *SLC37A1* [8], and *MGST1* [9]. Recently, as part of work presented elsewhere [10], we performed a genome-wide association analysis for milk volume in 4982 mixed breed cattle using a BayesB model [11, 12] and a panel of 3695 variants selected as tag-SNPs representing expression QTL (eQTL) from lactating mammary tissue. Of the top three loci explaining the greatest proportion of genetic variance in this model, genes representing the top and second to top effects have been well described for their role in milk production (*DGAT1* and *MGST1* respectively [5, 9]), whereas no causative gene appears to have been definitively assigned for the third signal on chromosome 5 between 75 and 76 Mbp.

This locus broadly overlaps QTL that were reported previously for milk yield [3, 13], milk protein yield [3, 13], milk protein concentration [1, 2, 14], and milk fat concentration [2, 9]. Although no gene has been definitively implicated, Pausch et al. [2] noted significant markers that were located adjacent to the *CSF2RB*, *NCF4*, and *TST* genes, and proposed the latter as the most likely candidate based on its proximity to the top associated variant. Other studies have proposed *CSF2RB* due to its high level of expression in the mammary gland [1, 14], or involvement in the JAK-STAT signalling pathway [3, 13]. Other nearby genes that have been suggested to cause these effects also include *MYH9* [3] and *NCF4* [13].

Given these observations, and the magnitude and diversity of effects at this locus, the aim of this study was to investigate this region on chromosome 5 in detail. By combining information on milk yield and composition with gene expression data from a large bovine mammary RNA sequence dataset, we highlight multiple lactation, gene expression, and RNA-editing QTL that segregate at this locus, and present *CSF2RB* as the most likely causative gene responsible for these effects.

## Methods

### Genotyping and phenotyping

All cows that had been genotyped using the Geneseek Genomic Profiler (GGP) LDv3 or LDv4 chips, and for which herd test phenotypes were available, were targeted in the current study (N = 29,350). These animals were selected because, based on preliminary sequence-based association analyses not reported here, these panels had been enriched with 365 polymorphisms identified as tag-variants of the chromosome 5 lactation QTL (spanning a region from 74.8 to 76.2 Mbp; [see Additional file 1]). These variants included 30 SNPs from the Illumina BovineSNP50 chip (50 k), which were added to assist with imputation by increasing the overlap between the GGP and 50 k panels. Tag-variants were targeted as custom content using a scheme that attempted to genotype sites in both orientations (two primers per site), resulting in 341 custom markers on the LDv3 chip, and 342 on the LDv4 chip for this locus. The breed composition of the animals used for these analyses comprised 8930 HF, 3599 J, and 15,652 HF × J cows, for which breed proportion was based on pedigree records, and purebreds were defined as animals that had a breed proportion higher than 13/16. We also included 1169 cows with minor proportions of Ayrshire ancestry.

Phenotypes were calculated from animal herd-test records for the three yield traits plus fat and protein concentration in milk. These phenotypes were generated using herd-test data from the first lactation, adjusted by using an ASReml-R [15] model with birth year, age at calving, breed, and heterosis as linear covariates, stage of lactation as a fixed effect, season/herd as an absorbed fixed effect, and animal as a random effect. Herd test records were sampled using Fourier-transform infrared spectroscopy on a combination of Milkoscan FT6000 (FOSS, Hillerød, Denmark) and Bentley FTS (Bentley, Chaska, USA) instruments.

### Imputation and association analyses

Genotypes for 29,350 animals were imputed to whole-genome sequence (WGS) resolution in the window of interest using Beagle 4 [16] as described previously [9, 17]. Briefly, a reference population of 565 animals, comprising Holstein-Friesians, Jerseys, and crossbred cattle, was sequenced using the Illumina HiSeq 2000 instrument to yield 100-bp reads. Read mapping to the UMD 3.1 bovine reference genome was conducted using the BWA MEM 0.7.8 software [18], followed by variant calling using GATK HaplotypeCaller 3.2 [19]. Variants were phased using Beagle 4 [16], and those with poor phasing metrics (allelic R^2^ < 0.95) were excluded, yielding 12,867 variants. Quality control filtering to remove variants with a MAF lower than 0.01% (N = 673) or Hardy–Weinberg equilibrium p-values below 1 × 10^−30^ (N = 461) resulted in a final set of 11,733 variants. As described above, the imputation window was enriched for custom, physically genotyped variants on the GGP-LDv3/4 chips, markedly increasing the scaffold density at this location.

Imputed genotypes for 639,822 autosomal SNPs on the Illumina BovineHD SNPchip were used to calculate a genomic relationship matrix (GRM) for the 29,350 animals of interest, using GCTA (version 1.91.3beta) [20, 21]. The imputation step also used the Beagle 4 software, leveraging a BovineHD-genotyped reference population of 3389 animals. Heritabilities for all phenotypes were calculated using this GRM with the REML option in GCTA. A leave-one-chromosome-out (LOCO) GRM was also created excluding chromosome 5, and used in combination with the imputed variant set and phenotypes to perform a mixed linear model analysis (MLMA-LOCO) [22] using GCTA.

### RNAseq, gene expression and eQTL

RNAseq data from lactating mammary gland biopsies representing 357 mixed-breed cows were generated as described previously [23]. Briefly, samples were sequenced using Illumina HiSeq 2000 instruments, yielding 100-bp paired-end reads. These were mapped to the UMD 3.1 reference genome using TopHat2 (version 2.0.11) [24]. The Stringtie software (version 1.2.4) [25] was used to quantify gene expression values for genes mapping to the window chr5:75–76 Mbp, yielding fragments per kilobase of transcript per million mapped reads (FPKM) and transcripts per million (TPM) [26] metrics. These calculations used gene models defined by the Ensembl gene build (release 81). Gene expression levels were also processed using the variance-stabilising transformation (VST) function implemented in the Bioconductor package DESeq (version 1.28.0) [27] to produce expression data suitable for analysis using linear models. The 357 biopsied cows comprised, using the same breed definitions as above, 224 HF, 3 J, and 126 HF × J cows, with the remaining four cows having minor proportions of Ayrshire ancestry.

WGS-resolution genotypes were imputed using the same WGS sequence reference described above in conjunction with a mixture of genotype panels (see Methods in [23]) for the 357 cows, yielding 12,825 variants in the 74.6–76.2 Mbp window. Removal of variants with more than 5% missing genotypes (N = 36) or a MAF lower than 0.5% (N = 1643) resulted in a final set containing 11,146 variants. VST-transformed gene expressions were analysed for genes with FPKM > 0.1, using the GCTA MLMA-LOCO method described above. The GRM was calculated using physically genotyped variants from the BovineHD SNP chip for 337 cows, and imputed BovineHD genotypes for the remaining 20 cows based on an Illumina SNP50 platform scaffold.

### RNA-editing site discovery and edQTL

RNA editing in the 3′-UTR of the *CSF2RB* gene was investigated in the discovery set of nine animals from [28], these animals having been previously sequenced using both RNAseq and WGS methodologies. Editing sites were identified using custom scripts [28] and by manual inspection of WGS and RNAseq BAM files for each animal. Sites were considered to represent RNA edits where: (1) an A-to-G variant was present in the RNAseq reads, but was absent from the WGS reads, and (2) had at least five reads containing ‘G’ at the position in every animal. This yielded 38 candidate edited sites. Following the recommendations of Ramaswami et al. [29] for non-*Alu* sites, the 38 candidate sites were examined for the presence of 5′ mismatches, simple repeats, homopolymer runs ≥ 5 bp, or splice junctions within 4 bp; however, none of the candidates were impacted by these filters, and all 38 were retained for further analyses.

Having determined the positions of variant sites, the rate of editing at each site was quantified in the larger ‘quantification set’ of 353 cows [28] with RNA editing phenotypes for each site generated by transforming editing proportions using the logit function. RNA editing QTL discovery was performed using these phenotypes by performing MLMA-LOCO, incorporating the same GRM and imputed WGS genotypes used for eQTL discovery (N = 353 animals).

RNA secondary structure around the edited sites was predicted using dot-plots as described by [28]. The sequence that contained all 38 edited sites and an additional 800 bp upstream and downstream was extracted and then plotted against its complement, with dots placed where at least 11 of 15 nucleotides surrounding a point were complementary. Diagonal lines in the resulting plot indicate regions of extended complementarity, which therefore have the potential to form double-stranded secondary structures.

### Copy number variant genotyping and imputation

Manual examination of the WGS BAM files suggested the presence of a copy-number variant (CNV) located downstream of *CSF2RB*, mapping to chr5:75,781,300–75,782,800. Copy numbers were estimated from WGS reads for each of the 560 cattle using the software package CNVnator (version 0.3) [30], based on sequence read depth. Thresholds for genotype calling of the CNV were set based on the histogram of the trimodal distribution of the copy number (CN) estimates, with a homozygous deletion being called when CN < 0.95, heterozygous 0.95 ≤ CN < 1.95, and homozygous wild type when CN ≥ 1.95. CNV genotypes were imputed into a larger population (N = 29,350), for use in association analyses, using Beagle version 4.1 [31], and the reference population of 560 cattle described above. Combining the reference genotype calls with the imputed population yielded a set of 31,950 animals for use in MLMA-LOCO analyses, as described above.

## Results

### Sequence-based association analysis at the chr5 interval

**Fig. 1:**
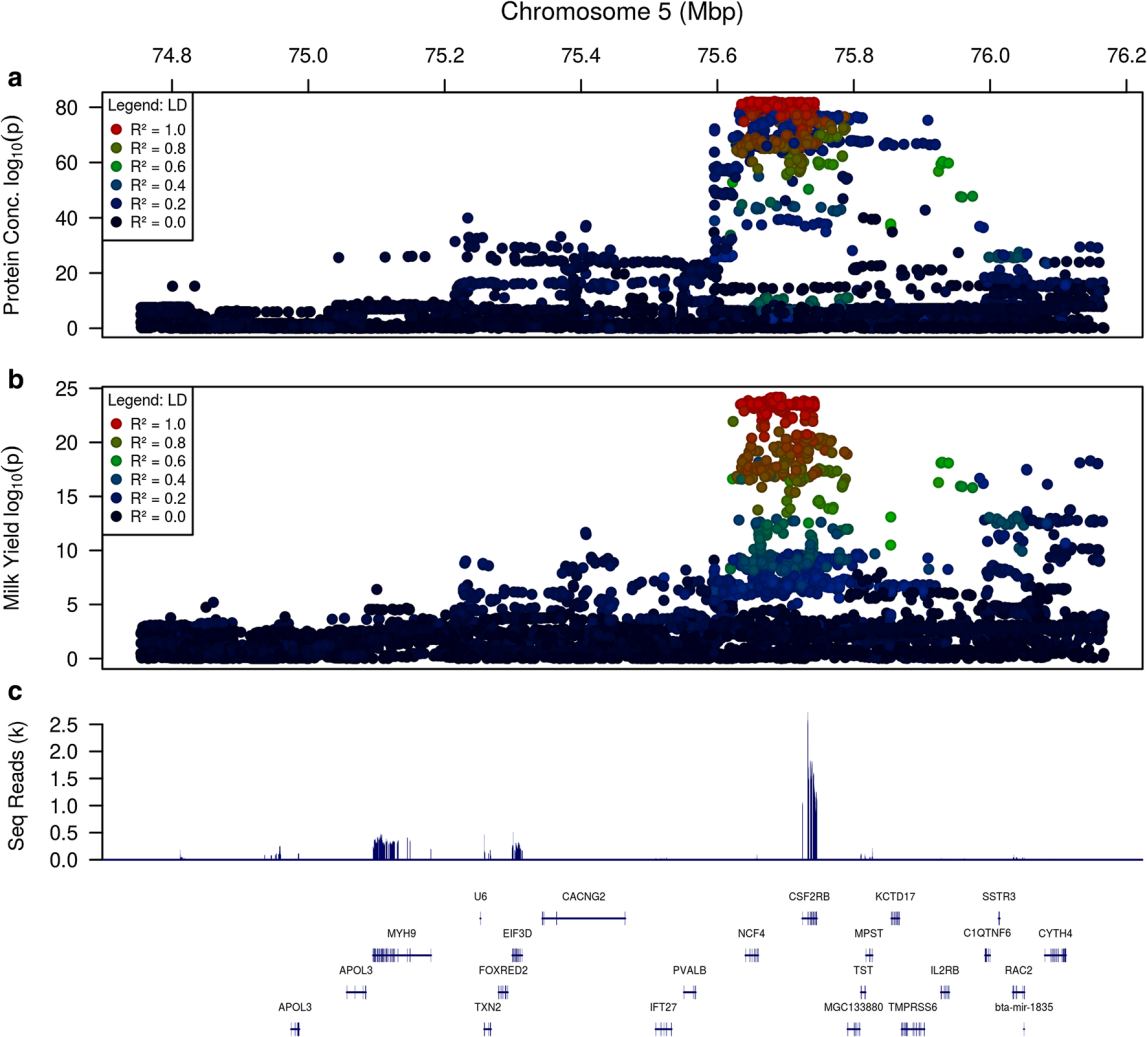
The genetic context of milk trait QTL. Panels **a** and **b**: QTL for the herd-test-derived phenotypes protein concentration (**a**) and milk yield (**b**). Colours represent LD (R^2^) with the most significant marker on a continuous scale, with colours provided in the legend for every 0.2 R^2^. Panel **c** shows the locations of genes mapping into this window (bottom) and the numbers of RNAseq reads mapping at positions across the window (top)

Fine mapping of milk yield and protein concentration QTL at the chr5:75–76 Mbp locus was performed using imputed sequence genotypes (see Methods) representing 29,350 cows. Sequence data were imputed using Beagle4 [16] (74.8–76.2 Mbp; 11,733 markers), and phenotypes were produced from herd-test records (N = 29,350 cows) from the animals’ first lactations to derive values for milk yield (MY), protein yield (PY), fat yield (FY), protein concentration (PC), and fat concentration (FC; see Methods). Mixed linear model association (MLMA) analyses were conducted using GCTA (version 1.91.3beta) [21]. The top associated variant for each of the five phenotypes is in Table 1. All QTL were significant at the genome-wide threshold 5 × 10^−8^. The most significant QTL was identified for PC, followed by FC and MY, and the least significant QTL was detected for FY. QTL for PC and MY are illustrated in Fig. 1.

**Table 1:** Top variants for milk yield and composition trait QTL

Phenotype	Top variant	Location	Gene	Beta	SE	P
FY (kg/day)	rs466308089	75,957,201	*IL2RB*	− 0.015	0.003	2.40 × 10^−8^
PY (kg/day)	rs108985709	76,157,976	*ELFN2*	0.004	0.001	1.05 × 10^−8^
MY (L/day)	rs208473130	75,685,770	*NCF4*	0.216	0.021	6.64 × 10^−25^
FC (%)	rs379739117	75,786,436	*RPL7*	− 0.055	0.004	3.27 × 10^−41^
PC (%)	rs208375076	75,651,326	*NCF4*	− 0.035	0.002	7.28 × 10^−83^

Phenotypes are daily yields for fat (FY), protein (PY), and milk (MY); and composition (percentage) phenotypes for fat (FC) and protein (PC). Locations on chromosome 5 are shown for the UMD 3.1 reference genome. The gene column indicates the nearest gene annotated in the UCSC genome browser for the bosTau8 assembly

**Fig. 2:**
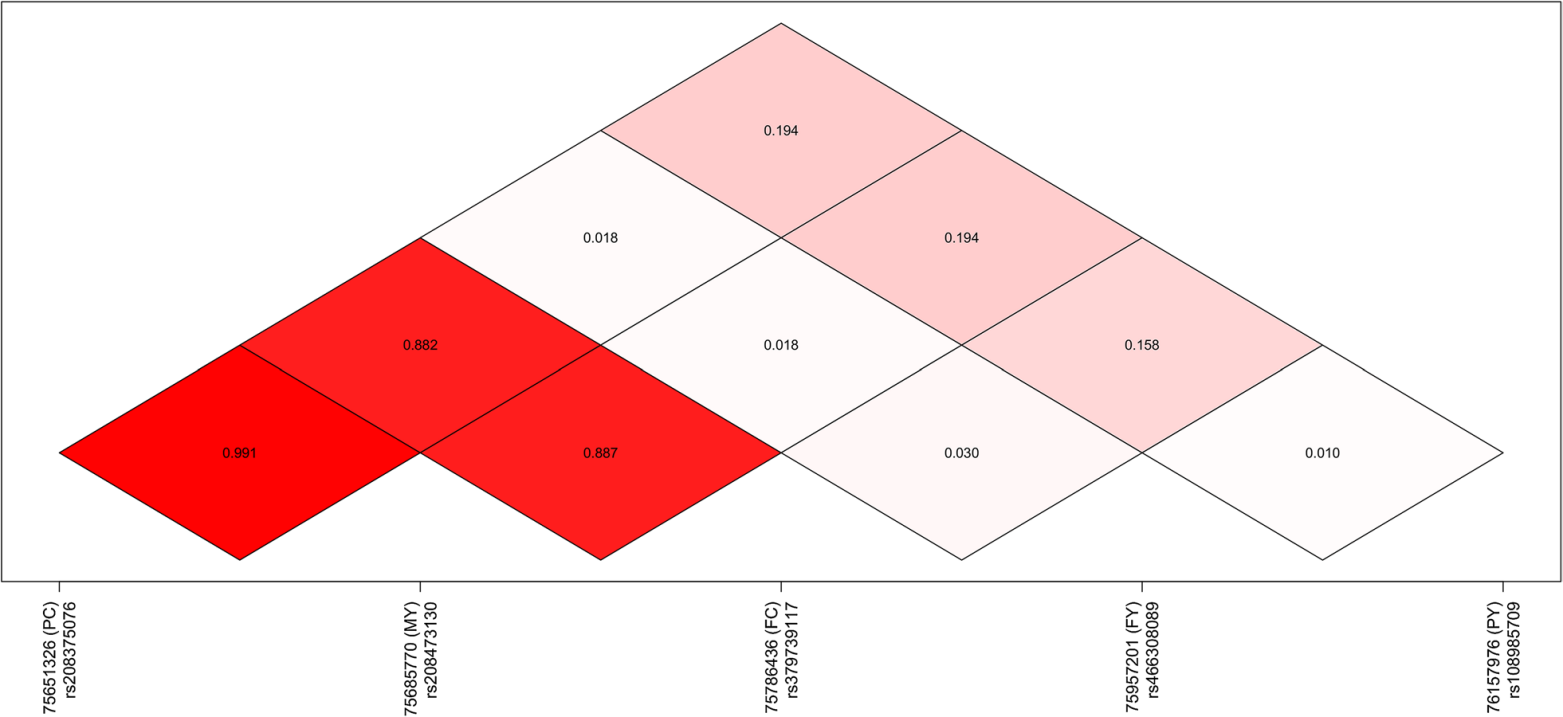
Linkage disequilibrium (LD) observed between the top associated markers for each phenotype (*R*^2^). Markers are identified using dbSNP reference SNP ID numbers. Phenotypes are as in Table 2

AI-REML analysis was performed, using a GRM calculated over all the autosomes, to estimate genomic heritabilities (*h*^2^; Table 2). To investigate these QTL further, the linkage disequilibrium (LD) statistics (*R*^2^) between each pair of top variants were calculated (Fig. 2). Strong LD was observed between the top variants for MY, FC, and PC (MY vs FC tag variants *R*^2^ = 0.887; MY vs PC tag variants *R*^2^ = 0.991).

**Table 2:** Genomic heritability estimates for milk yield and composition phenotypes

FY (kg/day)	FC (%)	PY (kg/day)	PC (%)	MY (L/day)
0.184 ±0.008	0.622 ±0.007	0.183 ±0.008	0.614 ±0.007	0.263 ±0.008

Phenotypes are milk fat daily yield (kg) and concentration (%), protein daily yield and concentration, and milk daily volume (L)

### Functional prediction of variant effects suggests regulatory QTL mechanisms

To assess potential functional effects of the statistically implicated QTL variants, all polymorphisms in strong LD (*R*^2^ >0.9) with the top-ranked QTL variants for each trait were extracted (N = 365 variants), and analysed using the Ensembl Variant Effect Predictor (VEP) [32]. Most of these variants (N = 247) were predicted to map outside of genes, whereas 113 were predicted to be intronic, with 58 in transcript ENSBTAT00000009911.4 (*NCF4*) and 55 in ENSBTAT00000011947.5 (*CSF2RB*). The remaining five variants were predicted to be synonymous mutations, with two in ENSBTAP00000009911.4 (*NCF4*) at positions p.Gln145 = and p.Tyr243 = , and three in ENSBTAP00000011947.5 (*CSF2RB*) at positions p.Asn58 = , p.Tyr405 = , and p.Glu424 = . Importantly, none of the highly associated variants were predicted to change the protein sequences of genes, suggesting a regulatory effect as the likely mechanism(s) of the QTL.

### Expression QTL analysis highlights three genes differentially expressed by genotype

To look for *cis*-eQTL effects that might explain the lactation QTL, gene expression levels were calculated for genes in the chr5:75–76 Mbp window, using RNAseq data representing lactating mammary tissue biopsies from 357 cows (Fig. 1c). Expression levels in FPKM and TPM were calculated using Stringtie (version 1.2.4) [25] and are in Table 3 for transcripts for which FPKM was higher than 0.1. The gene with the highest expression level was *CSF2RB*, which is consistent with previous observations in murine mammary RNAseq data [33]. Moderate expression was also observed for the candidate gene *MYH9*. However, the expression level of *NCF4* was very low, at FPKM = 0.406. The highest correlation between pairs of gene expression levels was observed for *TST* and *MPST* (*r *= 0.545 ±0.077), which is concordant with the published observation of a shared bidirectional promoter for these two genes [34].

**Table 3:** Median gene expression levels and top variants identified in eQTL analyses

Gene	Ensembl	FPKM	TPM	Top variant	MAF	Beta	SE	P
*APOL3*	ENSBTAG00000040244	0.934	1.166	rs433710540	0.101	0.128	0.0315	4.84 × 10^−5^
*CSF2RB*	ENSBTAG00000009064	61.888	80.081	rs384734208	0.439	0.428	0.0401	1.33 × 10^−26^
*EIF3D*	ENSBTAG00000001988	9.139	11.461	rs110614216	0.353	− 0.072	0.0138	1.66 × 10^−7^
*FOXRED2*	ENSBTAG00000000015	0.142	0.179	rs385243246	0.176	0.036	0.0133	6.52 × 10^−3^
*IFT27*	ENSBTAG00000026657	0.904	1.107	rs110654851	0.440	0.046	0.0103	8.01 × 10^−6^
*IL2RB*	ENSBTAG00000016345	0.285	0.359	rs43436480	0.364	0.058	0.0184	1.61 × 10^−3^
*MPST*	ENSBTAG00000030648	1.564	1.957	rs109488885	0.314	− 0.053	0.0144	2.40 × 10^−4^
*MYH9*	ENSBTAG00000010402	14.448	17.497	rs377857213	0.034	0.280	0.0715	9.07 × 10^−5^
*NCF4*	ENSBTAG00000007531	0.406	0.513	rs209273109	0.443	0.137	0.0168	4.30 × 10^−16^
*TST*	ENSBTAG00000030650	2.131	2.662	rs109922126	0.073	− 0.152	0.0313	1.19 × 10^−6^
*TXN2*	ENSBTAG00000000014	4.345	5.653	rs109450151	0.454	− 0.080	0.0116	5.85 × 10^−12^

Genes with FPKM values less than 0.1 are not shown. Gene symbols are from VGNC and Ensembl. Beta is the effect size of the minor allele on gene expression, measured in VST-transformed units. Three genes have eQTL which exceed the genome-wide significance threshold 5 ×10^−8^ [60]

**Fig. 3:**
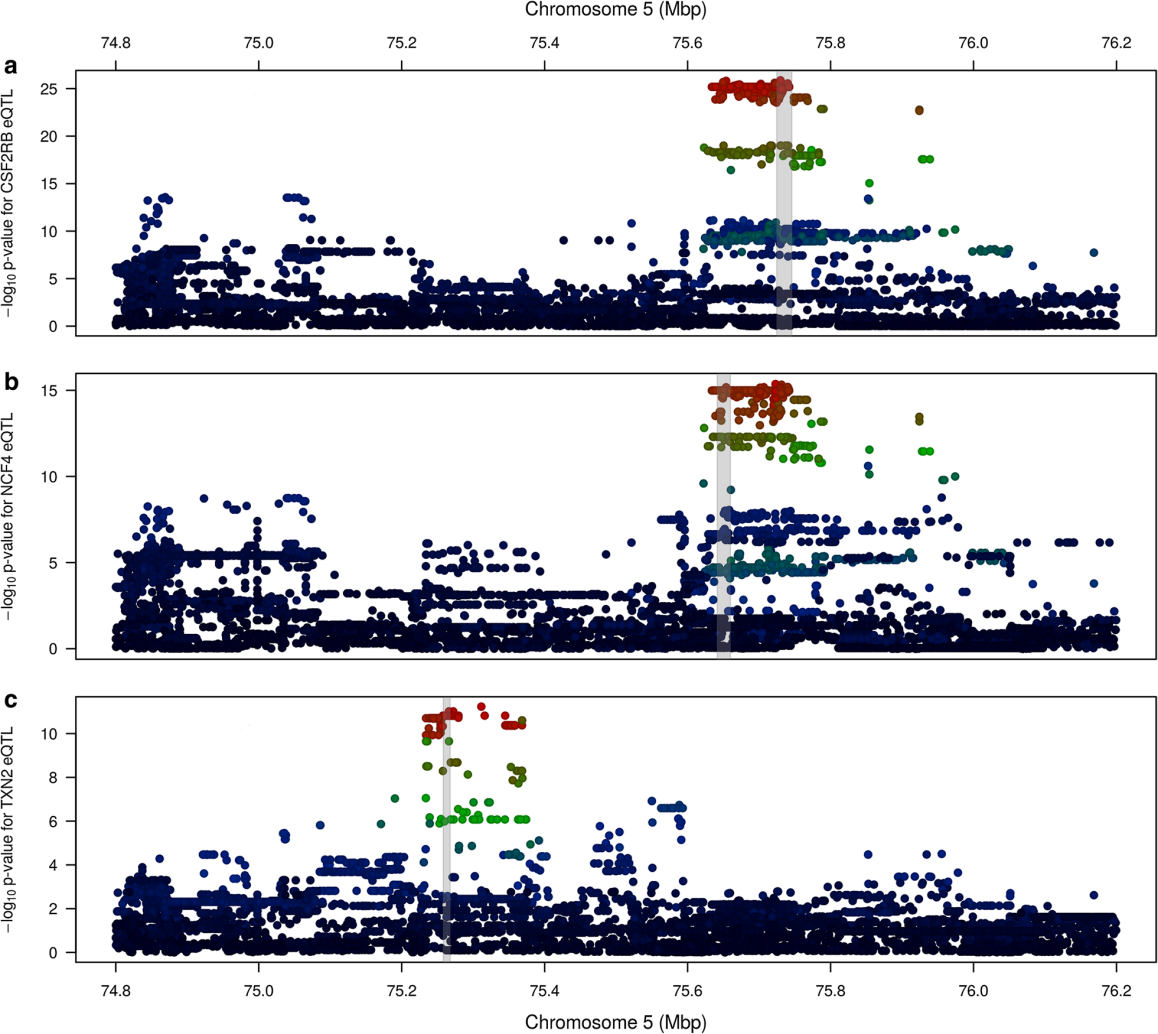
QTL plots showing eQTL for the three genes that exhibit genome-wide significant *cis*-eQTL (Table 3). From top to bottom, the three genes are **a** *CSF2RB*, **b** *NCF4*, and **c** *TXN2*. Colours represent correlations for each marker with the top variant for that eQTL (see Fig. 1 for legend). Grey bands indicate the location of the gene for which the eQTL is displayed

Association mapping was conducted for the 11 expressed genes in Table 3. To this end, gene expression data were first scaled using the variance-stabilising transformation (VST) implemented in DESeq (version 1.28.0) [27]. A GRM was then calculated for the 357 cows representing the RNAseq dataset, and the MLMA-LOCO method was performed as described for the analysis of lactation traits. This yielded genome-wide significant eQTL for three genes: *CSF2RB* (1.33 × 10^−26^), *NCF4* (4.30 × 10^−16^), and *TXN2* (5.85 × 10^−12^) (see Table 3 and Fig. 3). All three genes were located within the peaks of their respective eQTL, demonstrating regulation in *cis*.

**Fig. 4:**
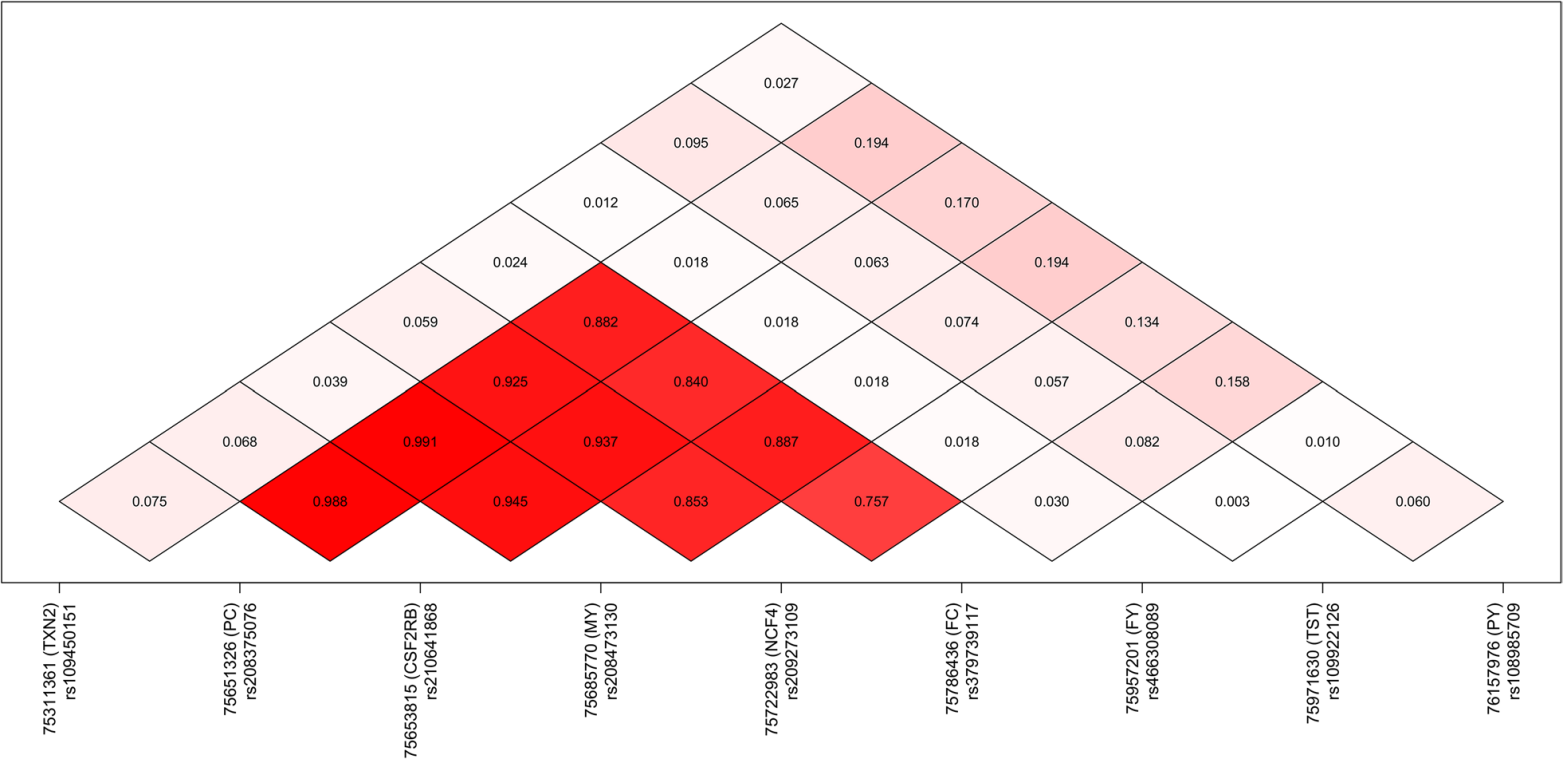
Linkage disequilibrium between the top tag variants for milk trait QTL and co-located gene expression QTL. Three genes with significant (P < 5×10^−8^) eQTL are included, along with the *TST* [2] that have previously been proposed as a candidate causative gene at this locus

In cases in which genetic regulation of gene expression (i.e., an eQTL) underlies a complex trait QTL, we expect that both QTL share similar association signals, with the most (and least) associated variants similar between phenotypes. To test whether any of the 11 expressed genes shared similarities with the milk QTL, Pearson correlations between the log_10_ p-values for each of the milk QTL and eQTL were calculated. Table 4 shows the QTL:eQTL correlations for all five phenotypes with three significant eQTL, plus the *TST* gene, which did not yield a genome-wide significant eQTL, although it has been proposed as a candidate underlying this locus. The eQTL for *CSF2RB* has *R*^2^ >0.5 (*r *>0.707) with three of the five milk phenotypes, while correlations for the neighbouring gene *NCF4* are just below this level. Neither of the *TXN2* or *TST* genes exhibited high correlations with any milk QTL. The eQTL for *CSF2RB* was also highly correlated with the *NCF4* eQTL (*r *= 0.863 ±0.005). A similar picture is obtained when examining the LD between the top tag markers for each QTL, with high LD observed (Fig. 4) among the tags for MY, FC, and PC with the tags for the *CSF2RB* and *NCF4* eQTL.

**Table 4:** Correlations between the − log_10_ p-values for milk trait QTL and co-located eQTL

Phenotype	*CSF2RB*	*NCF4*	*TST*	*TXN2*
FY (kg/day)	0.376 ± 0.017	0.164 ± 0.019	0.293 ± 0.018	0.024 ± 0.020
PY (kg/day)	0.562 ± 0.014	0.404 ± 0.017	0.425 ± 0.016	0.032 ± 0.020
MY (L/day)	0.849 ± 0.006	0.682 ± 0.011	0.306 ± 0.018	− 0.039 ± 0.020
FC (%)	0.756 ± 0.009	0.648 ± 0.012	0.104 ± 0.020	− 0.128 ± 0.020
PC (%)	0.754 ± 0.009	0.689 ± 0.011	0.059 ± 0.020	− 0.118 ± 0.020

Pearson correlations are shown, with 95% confidence intervals. Three genes with significant (P < 5×10^−8^) eQTL are shown, along with the *TST* gene [2] that has previously been proposed as a candidate causative at this locus

### Evidence of multiple, differentially segregating QTL for milk yield and protein concentration

**Fig. 5:**
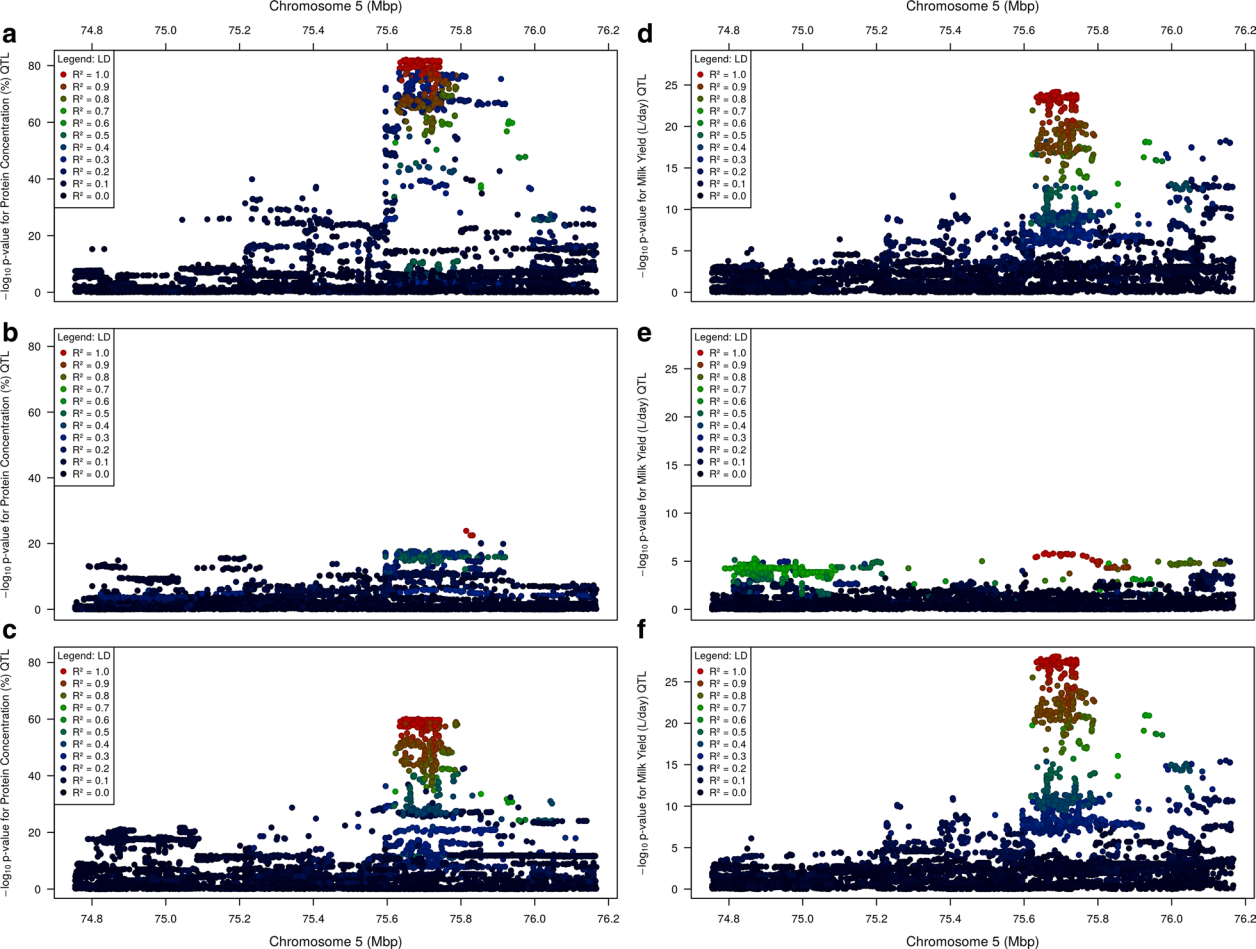
The effect of fitting the top variant on protein concentration (**a**–**c**) and milk yield (**d**–**f**) QTL. The top panels **a** and **d** show the QTL with no marker adjustments fitted; the centre panels **b** and **e** show the QTL after fitting the top variant from the panel above; and the bottom panels **c** and **f** show the QTL after fitting the top variant from the centre panel above. The phenotypes were adjusted by fitting the following markers: **b** rs208375076, **c** rs210293314, **d** rs208473130, **e** rs378861677

Examining Fig. 1a (repeated in Fig. 5a) suggested that protein concentration might be influenced by two co-located but mechanistically independent QTL, since a number of markers that are not in strong LD with the top marker nevertheless exhibit very small p-values (<1 × 10^−60^). To investigate this possibility, the top associated marker (rs208375076) was fitted as a fixed effect and the MLMA-LOCO analysis repeated using the residual, PC phenotype (Fig. 5b). The new top marker (rs210293314) remained highly significant (P = 1.30 × 10^−24^ after adjustment, 9.31 × 10^−41^ before adjustment), suggesting that it is tagging a different QTL. Adjusting the original protein concentration phenotype for rs210293314 and repeating the MLMA-LOCO analysis yielded the result shown in Fig. 5c. Here, the most significant marker was rs208086849, a variant that is largely statistically equivalent to the top rs208375076 marker from the original, unadjusted analysis (*R*^2^ = 0.999). These observations suggest the presence of two QTL for milk protein percentage.

This analysis was repeated with the MY phenotype (Fig. 5d). This phenotype showed little evidence of a second co-locating QTL, where fitting the top associated marker (rs208473130) dropped the signal below the genome-wide significance threshold (P = 1.36 × 10^−6^ for marker rs378861677; Fig. 5e). However, adjusting the MY phenotype by fitting rs378861677 and repeating the MLMA-LOCO analysis resulted in an increase in significance for the top marker rs208473130, from 6.64 × 10^−25^ to 8.63 × 10^−29^ (Fig. 5f), which suggests that there may indeed be an additional weak QTL, or the variant otherwise addresses some other confounding signal. The variants rs208086849 (from the PC analysis in the previous paragraph) and rs208473130 show very strong LD (*R*^2^ = 0.991), which suggests that both markers are in fact tagging the same QTL across PC and MY. In contrast, variants rs210293314 (PC analysis above) and rs378861677 show moderate to weak LD (*R*^2^ = 0.332), which suggests that the two signals tagged by these variants are genetically distinct.

Most of the QTL were represented by common tag-variants, with minor allele frequencies (MAF) higher than 0.4 across the whole population (Table 5). The two seemingly distinct PC QTL also segregated in both breeds, as do the PY and FC QTL. One of the two MY QTL, tagged by rs378861677, was uncommon in the Jersey population (MAF = 0.01), and the FY QTL tagged by rs466308089 had a MAF of only 0.002 in Jersey cows. The minor allele of the latter QTL was also the rarest signal in the population overall, with a MAF of 0.031.

**Table 5:** Minor allele frequencies for each top QTL variant

Phenotype	Variant	MAF All	HF (N = 8930)	J (N = 3599)	Cross (N = 15,652)
FY (kg/day)	rs466308089	0.031	0.042	0.002	0.032
PY (kg/day)	rs108985709	0.409	0.483	0.298	0.391
MY (L/day)	rs208473130	0.444	0.489	0.390	0.435
FC (%)	rs379739117	0.473	*0.476	0.391	0.464
PC (%)	rs208375076	0.446	0.492	0.391	0.437
MY (%)	rs378861677	0.116	0.182	0.010	0.101
PC (%)	rs210293314	0.276	0.333	0.184	0.264

Allele frequencies are shown across the entire study population of cows and by breed (*HF* Holstein–Friesian; *J* Jersey)

Asterisks (*) indicate breeds where the minor allele differs from that for the population as a whole. The markers listed in the top section of the table tag the primary QTL for each phenotype, while those listed in the bottom section tag secondary QTL

Since at least two differentially segregating QTL were detected at the locus, they may be underpinned by different genes and/or molecular mechanisms. To assess whether the significant, co-locating *CSF2RB* and *NCF4* eQTL were themselves comprised of multiple, overlapping signals (i.e. multiple *cis*-eQTL driven by different regulatory elements), the top associated variants were fitted as fixed effects to the gene expression phenotypes, and the analyses were rerun as above. This yielded new top markers with p-values of 8.87 × 10^−5^ and 1.75 × 10^−4^ respectively, suggesting that the expression of these two genes, if influenced by multiple regulatory factors, had weak effects, or were too heavily confounded by LD to differentiate clearly.

**Fig. 6:**
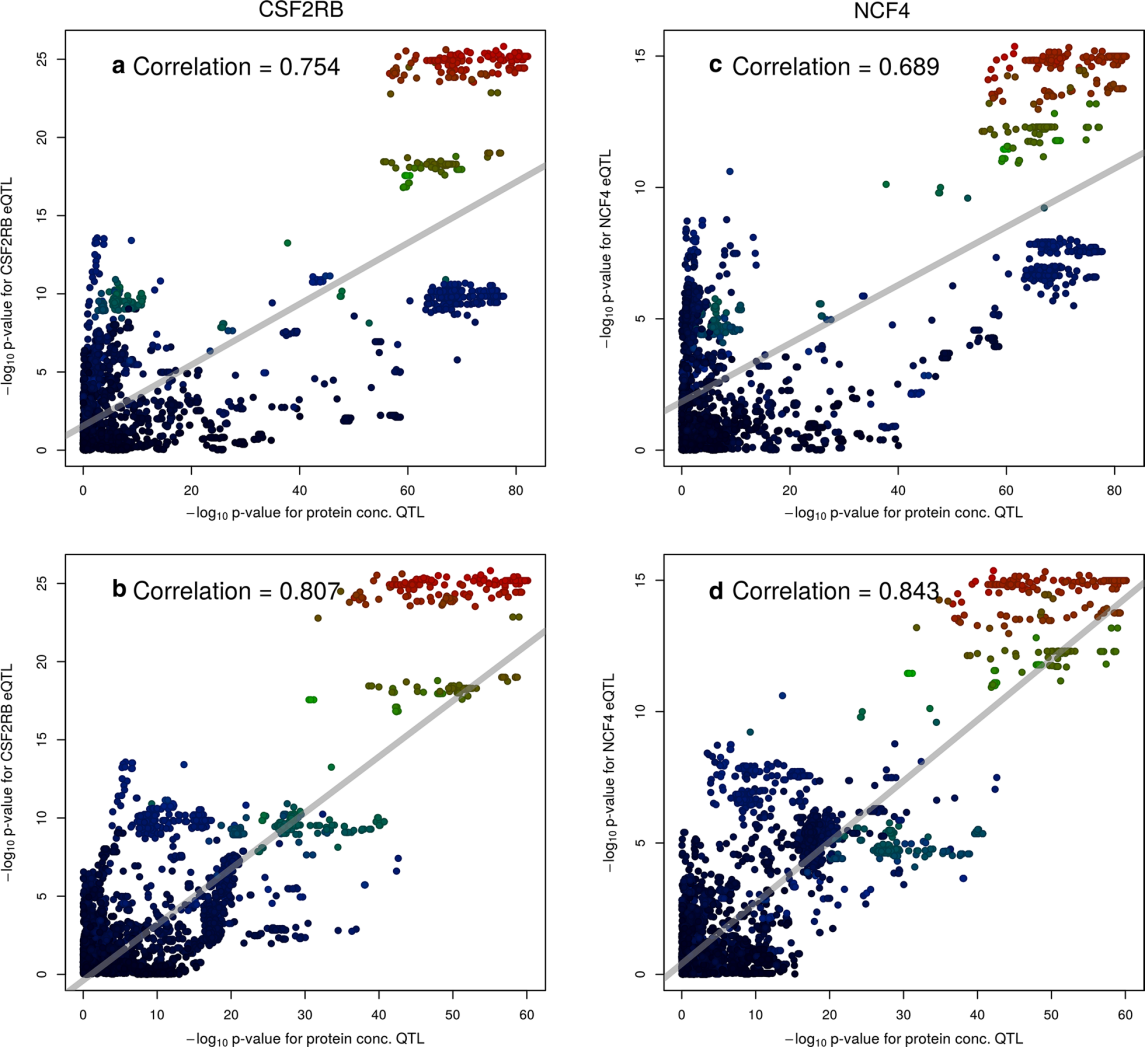
Correlations between eQTL and the co-located protein concentration QTL for the genes *CSF2RB* (left) and *NCF4* (right). Panels on the top row are plotted against the original protein concentration QTL (Fig. 5a), while panels on the bottom row are plotted against the phenotype after fitting rs210293314 (Fig. 5c)

To investigate how the eQTL might contribute to the multiple, co-locating PC QTL in comparative terms, the SNP-adjusted PC association results were used to calculate eQTL correlations, using the methodology described in the previous section. Notably, these analyses resulted in improved correlations with eQTL. The correlation between the *CSF2RB cis*-eQTL and the unadjusted PC phenotype was 0.754 ± 0.009 (Figs. 4, 6a). However, using the phenotype adjusted for rs210293314 yielded a correlation of 0.807 ±0.007 (Fig. 6b). The same pattern was observed for the *NCF4* gene, for which correlations improved from 0.689 ± 0.011 to 0.843 ± 0.006 (Fig. 6c, d). Applying the same approach to MY (unadjusted, and adjusted by rs37886167) similar results were obtained, albeit with only marginal increases: correlations with the *CSF2RB* eQTL increased from 0.849 ±0.006 to 0.872 ±0.005, and correlations with the *NCF4* eQTL increased from 0.681 ±0.011 to 0.713 ±0.010.

To investigate the possibility that secondary, co-locating PC and/or MY QTL might be caused by protein-coding variants, all variants in strong LD (*R*^2^ >0.9) with rs210293314 (secondary PC tag-SNP) or rs378861677 (secondary MY tag-SNP) were analysed using VEP as described previously. Of the 260 variants captured by this analysis, two missense SNPs were identified in conjunction with rs378861677, both mapping to exon 2 of *MPST*: rs211170554 (p.Asp129Asn) with a SIFT score of 0.88 (predicted tolerated), and rs209917448 (p.Arg47Cys) with a SIFT score of 0.01 (predicted deleterious). In the absence of additional eQTL that might account for the secondary PC and MY signals, these results suggest a potential protein-coding-based mechanism for the MY effect, at least.

### *CSF2RB* encodes a promiscuously RNA-edited transcript

**Fig. 7:**
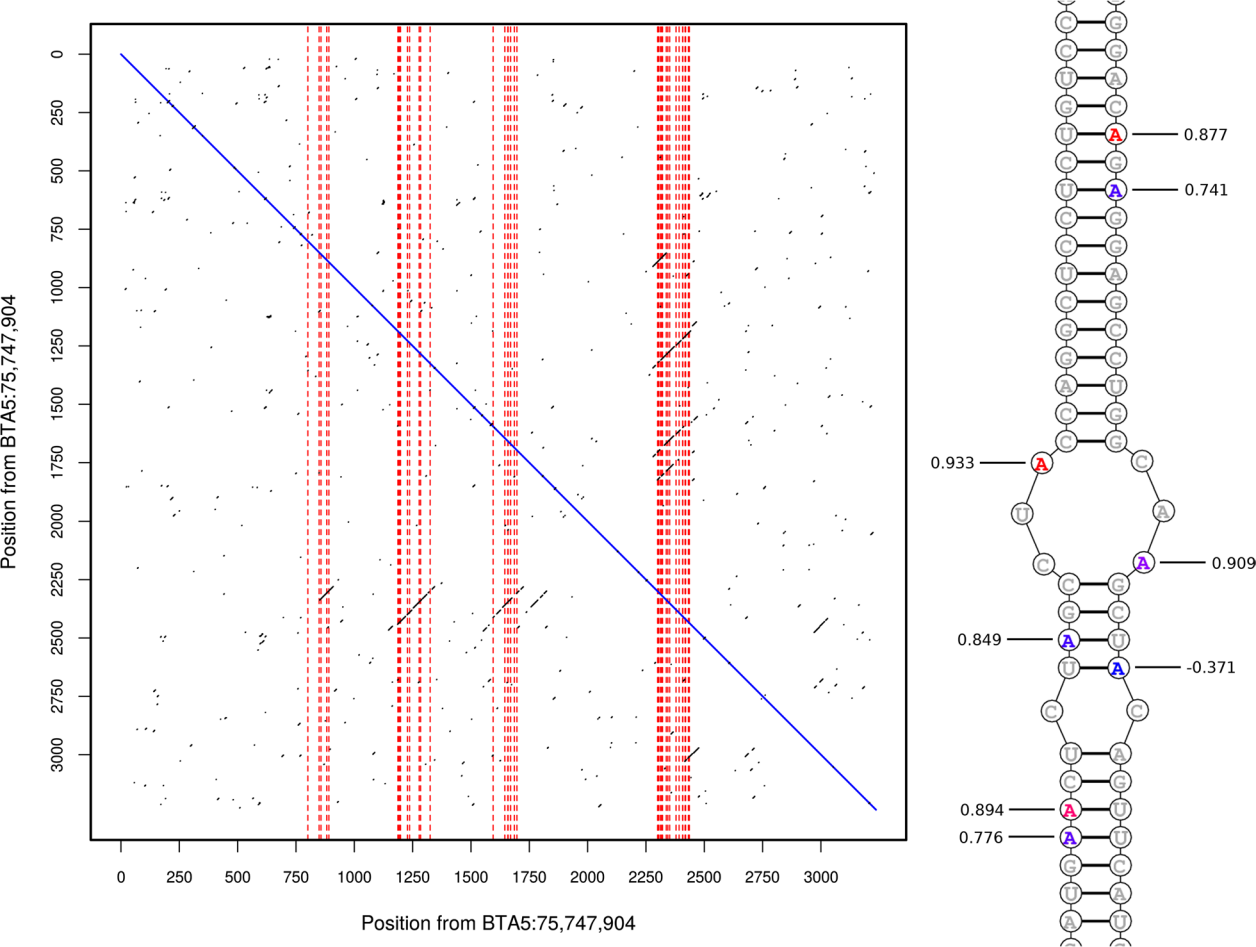
Left: dotplot of the sequence from the *CSF2RB* 3′-UTR against its complement. Positions are relative to chr5:75,747,904. Black dots indicate that seven of the 11 surrounding nucleotides are complementary. Vertical dashed red lines indicate the locations of predicted RNA-editing sites. Sections of the region 2275–2452 are complementary to the regions 837–915, 1178–1350, 1591–1719, and 1757–1832, suggesting that the UTR is able to fold into multiple configurations. Right: the section of predicted double stranded sequence between 1184 and 1217 on the left strand (running upward), and 2411–2444 on the right strand (running downward). Edited sites are coloured based on the strength of the edQTL at that site, from blue (not significant) to red (max P = 5.22 × 10^−26^). Sites are labelled with the correlation between the edQTL and the milk volume (MY) QTL after adjusting for marker rs208473130

Previous work [28] had identified four RNA editing sites that mapped to the introns of *CSF2RB*. Here, while manually examining RNAseq and WGS sequence reads mapping to the gene, a surprising number of additional RNA edits were observed (see Methods). This included 38 novel A-to-G variant sites present in the RNAseq data, yet absent from the whole-genome sequence representing the nine cows for which both data sources were available. These sites were present in four clusters within the 3′ UTR (Fig. 7), which had been missed from our previously published genome-wide analysis [28] because it was based only on reference annotations that failed to capture the full length 3′ UTR sequence, which was evident when empirically derived gene structures from the mammary RNAseq data were used. Because the ADAR enzymes responsible for adenosine-to-inosine editing (A-to-G in sequence reads) target double-stranded RNA [35, 36], we predicted the potential for the sequences around the edited sites to form double-stranded RNA. The dot-plot in Fig. 7 shows that, of the 38 edited sites (red dashed lines), 37 (97.4%) sit within regions of extended complementarity (diagonal black lines), thus having the potential to form double stranded secondary structures.

As recently reported, we have observed that a proportion of RNA-edited bases are genetically modulated for some sites [28]. To investigate potential genetic regulation of RNA-editing on *CSF2RB* transcripts, phenotypes for the proportion of reads edited were generated (see Methods), to detect RNA editing QTL (edQTL [28, 37]). Using the MLMA-LOCO method as applied for the eQTL analysis described above, genome-wide significant edQTL (P < 5 × 10^−8^) were identified for 18 of the 38 sites. Because RNA editing may impact gene expression by different mechanisms [38–40], we investigated whether any edQTL were correlated with the eQTL for *CSF2RB*. One site, mapping to chr5:75,750,220, had a correlation of 0.849 ±0.005 between the − log_10_ p-values of the edQTL and the eQTL. This edQTL was also strongly correlated with the *NCF4* eQTL (0.929 ±0.003).

As an extension to the hypothesis that edQTL might underlie changes in gene expression (i.e. eQTL), we reasoned that one or more of the milk phenotype QTL might also be impacted, as evidenced by the correlation values. Investigation of this hypothesis showed correlations *r* higher than 0.707 between edQTL and FC, PC, and PY (Table 6). In addition, we found very strong correlations (*r* > 0.9) between two edQTL (chr5:75,749,101 and chr5:75,750,335) and MY after adjusting for the genotype of marker rs208473130 (yield QTL illustrated in Fig. 5e, correlations in Fig. 7). A strong correlation (0.822) was also detected between the edQTL for chr5:75,748,760 and the PC QTL after adjusting for marker rs208375076 (PC QTL illustrated in Fig. 5b). As with the analyses of candidate protein-coding variants, these results suggest other alternative (and likely overlapping) mechanisms that may account for the multiple QTL segregating at the chromosome 5 locus.

**Table 6:** Correlations between the − log_10_ p-values for milk trait QTL and co-located edQTL

Phenotype	Edit site	Correlation
FC (%)	chr5:75,750,310	0.751 ±0.009
PC (%)	chr5:75,750,220	0.753 ±0.009
PC (%)	chr5:75,750,310	0.771 ±0.008
PY (kg/day)	chr5:75,748,794	0.799 ±0.007
PY (kg/day)	chr5:75,749,140	0.787 ±0.008
PY (kg/day)	chr5:75,750,204	0.718 ±0.010

Pearson correlations (with 95% confidence intervals) between the − log_10_ p-values for milk trait QTL and edQTL for sites mapping to the 3′-UTR of *CSF2RB*. Only sites and phenotypes where the correlation exceeded 0.707 (*R*^2^ >0.5) are shown

### Hypervariability at the *CSF2RB* locus presents an abundance of candidate causative variants

**Fig. 8:**
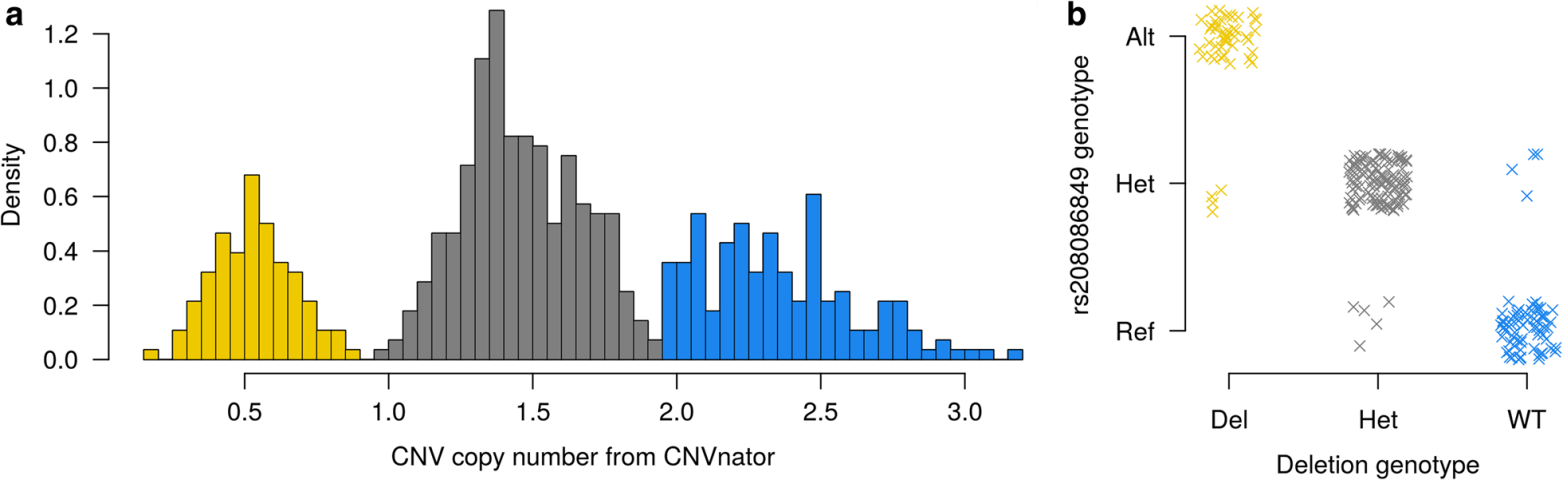
**a** Histogram of copy number genotype calls of 560 animals from CNVnator. Copy numbers follow a trimodal distribution, suggesting that the variant is bialleleic. Genotype classes are coloured in gold (homozygous deletion), grey (heterozygous) and blue (homozygous wild-type). **b** Deletion variant genotypes plotted against the genotypes of the rs208086849 variant. The two variants are in strong LD (R^2^ = 0.887). Points are jittered to increase visibility

Manual examination of the WGS alignments at the locus also revealed read depth anomalies at approximately chr5:75,781,300–75,782,800. This analysis revealed a suspected 1.5 kbp deletion variant, located between the *CSF2RB* and *TEX33* genes (downstream of the 3′ UTR of both genes given a ‘tail to tail’ orientation). To attempt to derive genotypes for this variant, the copy number at this site was estimated for 560 whole-genome sequenced cattle using CNVnator 0.3 [30]. The resulting estimates of copy number formed a trimodal distribution (Fig. 8a), which suggested a biallelic variant that could be assumed to be inherited in a Mendelian fashion [41]. Although one pseudogene maps to the region (LOC788541 60S ribosomal protein L7), the deleted segment appeared otherwise devoid of noteworthy genomic features.

To investigate the candidacy of the deletion as a potential causative variant for one or more of the QTL in the region, genotypes were called from CNVnator copy number predictions (see Methods), and the LD (R^2^) between the deletion and top QTL variants was investigated. Strong LD (0.887) was observed with the top markers for MY (rs208473130) and PC (rs208375076), as well as with rs208086849, the top variant for PC after adjusting for the secondary QTL (Fig. 8b). A slightly lower LD score was observed for FC (R^2^ = 0.807). The deletion allele was more frequent than the reference allele in the NZ dairy population (deletion = 0.547).

The strong LD between the ~ 1.5 kbp deletion and key QTL tag variants qualified the variant as a potential candidate for these QTL; therefore, we imputed the variant into the association analysis population to test for association directly. Using the same MLMA-LOCO analysis method that was applied for other variants, significant associations (P < 5 × 10^−8^) were observed for PC (P = 7.30 × 10^−71^), FC (P = 1.08 × 10^−30^), and MY (P = 1.18 × 10^−18^). Although highly significant, when ranking all variants by *p* value, the deletion variant never ranked higher than the 400th most significant marker; however, given the very large number of associated variants in this region generally (> 800 in the top 20 orders of magnitude for PC), and the fact that some of the read-depth-based genotype calls may be erroneous, the deletion remains a plausible candidate variant for future consideration of these QTL.

## Discussion

### Milk phenotype QTL

We report QTL mapping of a chromosome 5 locus for several milk yield and composition traits, with a diversity of gene expression and RNA editing QTL that could underpin these effects. We note, in particular, that some phenotypes exhibit multiple QTL that probably have distinct genetic causes. The FC and PC QTL are both in high LD with the MY QTL, which suggests that these effects may be mediated by changes in the total volume of milk produced without concomitant changes in fat or protein production. The fat and protein yield QTL are not in LD with either each other or with milk yield. However, these two QTL are less significant than the others by many orders of magnitude (see Table 1), which suggests that the lack of LD may be due to insufficient power in the dataset to identify reproducible tag variants. However, it should be noted that the MAF for the FY QTL is much lower than those for the MY and PY QTL, which suggests that this signal may indeed be discrete from the other two yield signals. The frequencies of the various tag variants across the breeds is also of note, which suggests that the QTL are both shared, and unique to individual breeds.

### Candidate causative genes

Several candidate causative genes have been previously proposed to underlie lactation effects at this locus, and based on the work presented here, we propose that one or both of the *CSF2RB* and *NCF4* genes are the likely candidates, with a predicted deleterious variant in the *MPST* gene also providing a potential candidate for milk yield QTL with a secondary effect.

The *CSF2RB* gene (ENSBTAG00000009064) encodes the common beta chain of the receptors for GM-CSF, interleukin-3, and interleukin-5, cytokines that are involved in regulating the proliferation and differentiation of hematopoietic cells [42]. The granulocyte–macrophage colony-stimulating factor (GM-CSF) is produced in the mammary gland by alveolar macrophages [43] where it enhances the bactericidal activity of milk neutrophils [44]. These receptors form a link in the JAK-STAT signalling pathway, operating via JAK2 and STAT5 [45]. The STAT5 proteins, especially STAT5A, are important for enabling mammopoiesis and lactogenesis [46, 47] and directly bind the gamma-interferon-activating sequence (GAS) found in the promoters of milk proteins such as beta-casein, [48], beta-lactoglobulin [49], and whey acidic protein in mice [49]. The importance of this pathway is further evidenced by associations with milk production traits observed at the *STAT5* locus [23, 50, 51]. Although the relevant ligands and subunits with which *CSF2RB* forms complexes are unknown in the current context, mutations that impact downstream interactions with STAT5 proteins could be assumed to impact milk production/composition phenotypes.

The *NCF4* gene (ENSBTAG00000007531) encodes neutrophil cytosolic factor 4, which forms the p40-phox subunit of the NADPH oxidase enzyme complex [52]. This enzyme produces superoxide (${\text{O}}_{2}^{ - }$), a reactive oxygen species produced in phagocytic cells during the respiratory burst [53], which is intended to kill invading fungi and bacteria [54]. *NCF4* has been shown to be upregulated in mastitic mammary glands [55], and two SNPs mapping to the *NCF4* gene have been associated with elevated somatic cell scores (SCS) [55, 56], a trait that is used as a surrogate phenotype for mastitis in dairy animals. Since cows suffering from mastitis produce smaller volumes of milk than healthy cows [57], this provides a possible mechanism by which *NCF4* could influence milk production. A more appealing mechanism is one that involves *CSF2RB* or *NCF4* but outside of a pathogen response context, given that the locus is better known for its impacts on milk production and composition in the absence of overt mammary infection.

Both the *CSF2RB* and *NCF4* eQTL were correlated with the MY QTL, with the former showing stronger correlations (*r *= 0.849 compared to 0.682). Lower correlations were observed between the two eQTL and the PC QTL (*r *= 0.754 and 0.691), however, removing one of the two apparent signals at this locus by fitting rs210293314 to the PC phenotype increased correlations for both candidate genes. Since no other genes showed similar patterns of co-association, we consider that one or both of these genes are the best candidates at this locus. The *CSF2RB* gene was expressed very strongly in mammary samples (TPM = 80.1), and by comparison, at a much higher level than *NCF4* (TPM = 0.51). This observation suggests a critical role for *CSF2RB*-mediated signalling in lactation, and given the plausible biological linkages of *CSF2RB* to these processes (via JAK-STAT signalling), we favour *CSF2RB* as the more likely of these two candidates.

The *TST* gene (ENSBTAG00000030650) was recently proposed by Pausch et al. [2] as a candidate for milk fat and protein percentage QTL at ~75–76 Mbp on chromosome 5. *TST* encodes thiosulfate sulfurtransferase, also known as rhodanese, a mitochondrial enzyme that catalyses the conversion of cyanide plus thiosulfate into thiocyanate plus sulfite [58]. It has been shown that the rhodanese enzyme (in misfolded form) can bind with 5S-rRNA, enabling its import into the mitochondria [59]. There appears to be limited literature implicating *TST* in mammary development and milk production, and given that the gene maps downstream of association peaks in our dataset, and has no prominent eQTL by which to mediate these effects, a role for this gene seems unlikely for QTL in the NZ population. This does not preclude the involvement of the gene in other populations, however, we consider that the most parsimonious hypothesis is that these QTL are shared across populations, at least partially underpinned by regulatory variants modulating the expression of the *CSF2RB* gene.

### RNA editing and edQTL

Previously, we [10] reported four RNA editing sites mapping to the *CSF2RB* gene, one of which (chr5:75,739,106) showed a significant edQTL (smallest P = 6.68 × 10^−13^). This site exhibited only modest correlations with the *CSF2RB* eQTL, or with the milk yield or composition QTL [10].

In the current paper, we report the discovery of 38 additional RNA-editing sites mapping to the 3′-UTR of *CSF2RB*. These sites were not identified in the previous work since they map approximately 3 kbp downstream of the gene structure based on the Ensembl reference annotation. Two of the novel sites, chr5:75,749,101 and chr5:75,750,335, exhibited edQTL with correlations exceeding 0.9 with the milk yield QTL after adjusting for marker rs208473130. The correlation between the *CSF2RB* eQTL and the same milk QTL was − 0.173, which suggests that, if the lactation effects indeed derive from an RNA-editing-based mechanism, this mechanism is not wholly reflected by the gene expression data used to quantify the eQTL effects.

## Conclusions

We have examined a previously implicated chromosome 5 locus for milk yield and composition traits, and identified highly significant QTL for milk yield, protein concentration, and fat concentration. Using a large mammary RNA sequence resource, we have conducted eQTL mapping of the locus and show that expression of *CSF2RB*, a highly expressed gene involved in signalling pathways that are important to mammary development and lactation, appears to be responsible for these effects. RNA editing sites were also discovered in the 3′-UTR of *CSF2RB*, and edQTL for two of these are correlated with one of two co-located but differentially segregating milk yield QTL, which was also in strong LD with a predicted deleterious missense variant in the *MPST* gene. These results highlight the pleiotropic nature of the *CSF2RB* gene, and showcase the mechanistic complexity of a locus that will require further statistical and functional dissection to catalogue the full multiplicity of effects.

## Additional file

**Additional file 1.** Polymorphisms targeted as custom content for the GGP LDv3 and LDv4 chips. These were identified as tag variants of chromosome 5 lactation QTL in the window 74.8–76.2 Mbp.
